# Homogenizing effect of PEEP on tidal volume distribution during neurally adjusted ventilatory assist: study of an animal model of acute respiratory distress syndrome

**DOI:** 10.1186/s12931-022-02228-x

**Published:** 2022-11-24

**Authors:** Hannes Widing, Elena Chiodaroli, Francesco Liggieri, Paola Sara Mariotti, Katarina Hallén, Gaetano Perchiazzi

**Affiliations:** 1 Hedenstierna Laboratory, Department of Surgical Sciences, Uppsala University, Akademiska Sjukhuset, Ing 40, 3 Tr, 751 85 Uppsala, Sweden; 2 Department of Anaesthesiology and Intensive Care Medicine, Region Västra Götaland, Sahlgrenska University Hospital/Östra, Gothenburg, Sweden; 3 Department of Anesthesia and Intensive Care, ASST Santi Paolo e Carlo, San Paolo University Hospital, Via Di Rudinì 8, Milan, Italy; 4 Division of Anesthesia and Intensive Care, San Martino Policlinic University Hospital, 16132 Genoa, Italy; 5 Department of Medical and Surgical Sciences, Anesthesia and Intensive Care Unit, University of Foggia, Foggia, Italy; 6 Department of Anesthesia, Operation and Intensive Care, Uppsala University Hospital, Uppsala, Sweden

**Keywords:** Positive end-expiratory pressure, PEEP, ARDS, NAVA, Assisted breathing, Spontaneous breathing

## Abstract

**Background:**

The physiological response and the potentially beneficial effects of positive end-expiratory pressure (PEEP) for lung protection and optimization of ventilation during spontaneous breathing in patients with acute respiratory distress syndrome (ARDS) are not fully understood. The aim of the study was to compare the effect of different PEEP levels on tidal volume distribution and on the ventilation of dependent lung region during neurally adjusted ventilatory assist (NAVA).

**Methods:**

ARDS-like lung injury was induced by using saline lavage in 10 anesthetized and spontaneously breathing farm-bred pigs. The animals were ventilated in NAVA modality and tidal volume distribution as well as dependent lung ventilation were assessed using electric impedance tomography during the application of PEEP levels from 0 to 15 cmH_2_0, in steps of 3 cmH_2_0. Tidal volume distribution and dependent fraction of ventilation were analysed using Wilcoxon signed rank test. Furthermore, airway, esophageal and transpulmonary pressure, as well as airway flow and delivered volume, were continuously measured during the assisted spontaneous breathing.

**Results:**

Increasing PEEP improved oxygenation and re-distributed tidal volume. Specifically, ventilation distribution changed from a predominant non-dependent to a more even distribution between non-dependent and dependent areas of the lung. Dependent fraction of ventilation reached 47 ± 9% at PEEP 9 cmH_2_0. Further increasing PEEP led to a predominant dependent ventilation.

**Conclusion:**

During assisted spontaneous breathing in this model of induced ARDS, PEEP modifies the distribution of ventilation and can achieve a homogenizing effect on its spatial arrangement. The study indicates that PEEP is an important factor during assisted spontaneous breathing and that EIT can be of valuable interest when titrating PEEP level during spontaneous breathing, by indicating the most homogeneous distribution of gas volumes throughout the PEEP spectrum.

## Background

The Acute Respiratory Distress Syndrome (ARDS) is associated with different derangements of lung physiology [1], such as inhomogeneity in tidal volume distribution and ventilation-perfusion (V/Q) mismatch, predominantly observed in dependent lung regions [2], and is marked by an elevated mortality [3].Tidal volume distribution inhomogeneities, deriving from local differences in time constants of the affected areas, may aggravate lung injury by developing high trans-alveolar strain forces [4] and potentially resulting in ventilator-induced lung injury (VILI) [5]. Moreover the atelectatic dependent lung region impedes optimal gas exchange, by the phenomena of low V/Q or shunting [6], and can be susceptible to the phenomena of tidal recruitment-derecruitment [7, 8].

The relation between positive end-expiratory pressure (PEEP) and these heterogeneous alterations is complex, additionally depending on the modality of ventilation that is implemented [9–11].

There is an ongoing debate about the use of assisted/spontaneous breathing (SB) modes in ARDS; as both positive effects, above all the counteracting of diaphragm atrophy [12], and potential drawbacks, the most detrimental being the patient self-inflict lung injury (P-SILI) [13]), exist. The optimal setting of PEEP is proposed to be one of the most important factors determining the risk of ventilator-induced lung injury (VILI) during SB [14].

Neurally Adjusted Ventilatory Assist (NAVA) is an assisted mode of ventilation that uses the electrical activity of the diaphragm (Edi) to control the timing and delivery of pressure from the ventilator, in relation to the frequency and amplitude of muscle activity of the diaphragm, indirectly representing phrenic nerve activity and central breathing center [15, 16]. NAVA is widely used, however not studied to the same extent as controlled or other assisted SB modes. Furthermore, the specific effects of PEEP on ventilation distribution during NAVA ventilation have not been studied thoroughly.

Thorax electrical impedance tomography (EIT) is a well-established imaging technique [17] based on measure of impedance (Z), whose changes (ΔZ) between end-expiration and end-inspiration, are linearly proportional to the tidal volume during mechanical ventilation [18, 19]. Moreover EIT yields topographic information about ventilation distribution in different settings [20–22].

One method to analyse the data derived from EIT, is to compute the dorsal fraction of ventilation (DFV), representing the proportion of tidal volume reaching dependent lung region in relation to total tidal volume, as proposed by Brochard et al. [23]. When DFV is 50%, ventilation can be considered homogeneously distributed between the dependent and nondependent portions of the lung [24, 25].

The aim of the present study was to test the hypothesis that increasing PEEP has a homogenizing effect on tidal volume distribution during NAVA, in an experimental animal model of ARDS.

## Methods

The study was approved by the local ethical board for animal studies, in Uppsala, Sweden (Approval number 58 18_20174_2017), and conducted according to the European Union directive 2010/63/EU, the Helsinki convention for the use and care of animals and the National Institute of Health Guidelines. The reporting of the experiment follows the ARRIVE guidelines [26] on the fair use of animals in research.

10 farm-bred pigs of both sexes (28.4 ± 2.1 kg) were premedicated using tiletamine-zolazepam (6 mgkg^−1^, Boehringer Ingelheim, Stockholm, Sweden) and xylazine (2.2 mgkg^−1^, Rompun Bayer, Leverkusen, Germany) after arriving to the laboratory, and placed supine. Canulation of an ear vein was performed and anesthesia was induced and maintained, using ketamine infusion (20 mgkg^−1^ h^−1^, Ketaminol, Vetpharma, Intervet, Stockholm, Sweden), allowing spontaneous breathing. Absent reaction to a painful stimulus at the hoof was used to ensure adequate depth of anesthesia. A surgical tracheostomy was performed and a tracheal tube (tube size 9, Mallinckrodt Pharmaceuticals, Athlone, Ireland) was inserted. Mechanical ventilation was initiated using Servo-i ventilator (Maquet Critical Care, Solna, Sweden) in pressure support ventilation, using PEEP 5 cmH_2_O, driving pressure of 10 cmH_2_O above PEEP and FiO_2_ of 0.5, during further instrumentation. A central venous catheter and a pulmonary artery catheter (PAC, 7.0 French, Swan-Ganz Thermodilution Catheter, Baxter, Irvine, CA, United States) were introduced via the femoral vein, using ultrasound guidance. An arterial catheter (20 G, Becton–Dickinson Critical Care Systems, Mississauga, ON, Canada) was placed in the femoral artery. Except for tracheostomy, all neck tissue were left intact, reducing interference with accessory breathing muscles. An EIT electrode belt (Size XXS, 32 electrodes, Timpel, Eindhoven, The Netherlands) was placed surrounding the thorax and connected to the EIT machine (Enlight, Timpel, Eindhoven, The Netherlands). The EIT belt was positioned at a level immediately caudal to the insertion of the forelegs on the chest wall and belt size was chosen in accordance to recommendations by the manufacturer. An esophageal balloon and a gastric balloon (esophageal catheter, Erich Jaeger GmbH, Höchberg, Germany) were inserted orally and placement was controlled using an occlusion technique, described by Baydur et al. [27]. Thereafter, a NAVA catheter (size 16F nasogastric catheter with multiple array electrodes) (Maquet, Solna, Stockholm, Sweden) was introduced orally and connected to the ventilator. Placement at the diaphragmatic dome was corrected in accordance to the guiding system provided by the ventilator, as described by Barwing et al. [28]. A chest x-ray scan was thereafter used to ensure the correct placement of the NAVA catheter and the balloons. Furthermore, central venous pressure (CVP), heart rate (HR), arterial blood pressure, pulmonary arterial blood pressure, blood temperature and transcutaneously measured oxygen saturation, by pulse oximetry (SpO_2_), were continuously measured and monitored (SC 9000 XL, Siemens Medical Systems Inc., Danvers, MA, United States). A solution of Ringer´s acetate was infused at 5 ml kg^−1^ h^−1^, maintaining fluid balance.

### Monitoring of respiratory mechanics

Pressure at airway opening (P_AO)_) together with esophageal (P_ESO_) and gastric pressure (P_GA_) were continuously recorded using dedicated transducers (DigimaClic Pressure Transducers, Special Instruments GmbH, Nördlingen, Germany). Airway flow (V̇) was monitored using a Fleisch pneumotachograph (Laminar Flow Element type PT, Special Instruments GmbH, Nördlingen, Germany) positioned at the endotracheal tube and connected to a differential pressure transducer (Diff-Cap Pressure Transducer, Special Instruments GmbH, Nördlingen, Germany). Measurements and signals were digitally converted using an analog-to-digital converter card (PowerLab 16/35, AD Instruments NZ Limited, Dunedin, New Zealand) and stored on a personal computer (Intel Centrino, Intel Corp., Santa Clara, CA, United States) at a sampling frequency of 200 Hz using LabChart Software (AD Instruments NZ Limited, Dunedin, New Zealand). Transpulmonary pressure (P_TP_) was monitored by a continuous calculation of the difference between P_AO_ and P_ESO_. By integrating V̇, inspired and expired volumes (V) were calculated. For Edi-signal recording, a serial cable was connected to the personal computer and the signal was sampled at a rate of 100 Hz, by the use of Servo-tracker V 4.0 software (Maquet Critical Care, Solna, Sweden).

### Induction of lung injury

After instrumentation, remifentanil infusion (0.25–0.5 µgkg^−1^ min^−1^, Remifentanil Orion, Orion Pharma, Espoo, Finland) was initiated and Rocuronium (20 mg, Rocuronium Fresenius Kabi 10 mg ml^−1^, Fresenius Kabi AB, Uppsala, Sweden) was administered to suppress breathing and movement during lung injury induction. Volume controlled ventilation, tidal volume of 8 ml kg^−1^, respiratory rate 30/min, PEEP 5 cmH_2_O, FiO_2_ 1.0, was initiated and a mild ARDS-like condition was induced by repeated lung lavages, using 30 mlkg^−1^ warm saline followed by airway suctioning. The injury-inducing process was repeated until a PaO_2_/FiO_2_ of ≤ 250 mmHg was reached and maintained after 10 min of mechanical ventilation at PEEP 5 cmH_2_O. Thereafter remifentanil infusion was stopped and SB was reestablished.

### NAVA ventilation and study protocol

After the return of spontaneous ventilation, NAVA level was titrated, in accordance to the method described by Brander et al. [29]. The NAVA level was thereafter not changed throughout the experiment and FiO_2_ was kept at 1.0. NAVA ventilation was continued for 20 min, allowing stabilization prior to the initiation of the study protocol. Thereafter, an incremental and decremental PEEP protocol was initiated. PEEP was increased stepwise from 0 cmH_2_O to 15 cmH_2_O in steps of 3 cmH_2_O and thereafter decreased again to 0 cmH_2_O in steps of 3 cmH_2_O. Hereby, the animals were used as their own controls, and all animals underwent the same treatment. The same order of PEEP level was used for all animals, allowing for evaluation of recruiting effect of the complete maneuver, when comparing incremental PEEP levels to decremental PEEP levels. For each PEEP level, settings were kept for 10 min prior to data acquisition, allowing establishment of steady state conditions. Thereafter, EIT scans (sampling rate: 50 Hz), Edi signal, ventilator parameters, airway flow and pressure readings, esophageal and gastric pressures were acquired continuously for 1 min. In addition, arterial blood gas samples were collected. Thereafter, the PEEP level was changed according to protocol and registrations were repeated after 10 min. After the study protocol, animals were euthanized using high dose potassium chloride.

### Data analysis

EIT data were analyzed using EIT main 8.11 (EIT analysis tool, Timpel, Eindhoven, The Netherlands). Tomographic scans were divided into four ROIs, labeled from the anterior to posterior as: anterior, mid-anterior, mid-posterior and posterior. Mean Z, relative to a machine-set zero, for each ROI was calculated. From the continuous EIT recording during non-interrupted spontaneous breathing, end-inspiratory and end-expiratory scans, for the first three breaths for each data acquisition, were identified and further analyzed. The difference between end-inspiratory and end-expiratory Z was calculated for each ROI respectively, representing ΔZ. In addition, the data was calculated in relation to total change in Z in all four ROIs, allowing for proportionate calculation of ΔZ. As ΔZ correlates linearly to change in volume, proportionate change in ΔZ was assumed to represent the relative distribution of tidal volume in each ROI. The two most anterior ROIs combined were presented as non-dependent region and the two most posterior ROIs combined were presented as dependent region.

Dependent fraction of ventilation was calculated as a percentage of total impedance change being distributed to the dependent regions, as of: *DFV (%): ∆Z dependent region / ∆Z whole lung*, as presented by Yoshida et al. [24] amongst others.

Airway pressure, airway flow and esophageal pressure during maximum transpulmonary pressure was identified and analyzed, using three representative breaths from each data acquisition. In addition, maximum airway flow and maximum airway pressure were obtained in each breath respectively.

### Respiratory mechanics

For each of these breaths, change in esophageal pressure (ΔP_ESO_) was calculated by subtracting the esophageal pressure P_ESO_ at end-expiration from the P_ESO_ at the point of maximum transpulmonary pressure (P_TP max tracing_), identified by transpulmonary pressure tracings. Maximum transpulmonary pressure (P_TP max_) was calculated by subtracting ΔP_ESO_ from airway pressure at P_TP max tracing_. As the pigs were breathing continuously and no inspiratory pause was used, transpulmonary pressure was corrected for the resistive component of the airway pressure, in order to obtain the true transpulmonary pressure acting on the alveoli.

By using the Multilinear fitting method [30] for P_TP_, V̇ and tidal volume, compliance (C) and resistance of the respiratory system (R_RS_) during each breath were calculated. By multiplying R_RS_ by the flow at P_TP max tracing_ (= V̇_TP max tracing_) of that same breath, the true maximum transpulmonary pressure (P_TP true max_) could be calculated using.

$${{\text{P}}_{\text{TP true max}}}\, = \,{\left( {{{\text{P}}_{{\text{AO}}}}\, - \,\Delta {{\text{P}}_{{\text{ESO}}}}} \right)_{\text{TP max tracing}}}\, - \,{{\text{R}}_{{\text{RS}}}} \times \,{\dot V_{\text{TP max tracing}}}$$

These calculations were repeated for each studied breath, as was done in previous studies [31].

### Statistics

Because ΔZ presented a non-normal distribution (Shapiro–Wilk test), we applied nonparametric tests. Wilcoxon signed rank test was used for comparing paired data and Mann Whitney test was used when comparing non-paired data. For all statistical methods, a p-value ≤ 0.05 was considered statistically significant. The dependency of ΔZ on PEEP in dependent and non-dependent lung region was also tested using linear regression analysis. All the other measurements presented a normal distribution and were analysed using student t-test (α = 0.05). To adjust for family-wise error rate when performing multiple comparison analysis, the Bonferroni correction was used for adjusting the α level.

## Results

All 10 animals survived the study protocol. For the study of ∆Z, 330 breaths and 660 EIT scans were analyzed. As for respiratory mechanics, 12 of the total 330 breaths analyzed were lost due to technical circumstances. Baseline data is presented in Table 1.

**Table 1:** Baseline data

Baseline characteristics	Mean (± SD)
Weight (kg)	28.4 ± 2.1
NAVA level (H_2_O μV^−1^)	2.3 ± 0.5
Sampled PEEP (cmH_2_O)	0.8 ± 0.6
Tidal volume (ml)	153 ± 36
Respiratory rate	75 ± 13
P_ESO_ (cmH_2_O)	− 2.5 ± 1.5
P_TP_ (cmH_2_O)	8.6 ± 3.2
PO_2_/FiO_2_ (kPa)	26 ± 14

Baseline data for all animals post injury

### Oxygenation and transpulmonary pressure

**Fig. 1:**
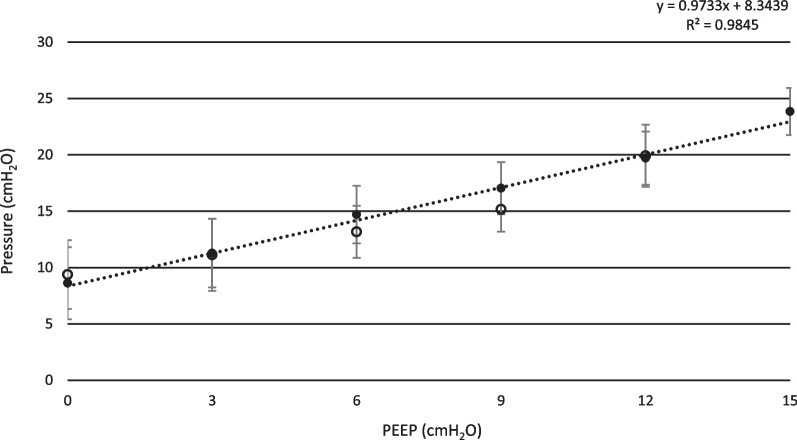
Transpulmonary pressure. Mean inspiratory transpulmonary pressure shown for both incremental and decremental phases of the PEEP protocol. Black dots represent incremental PEEP levels and transparent circles represent decremental PEEP levels. The inspiratory transpulmonary pressure is corrected for the resistive component of the airway pressure, using a multilinear fitting method. Standard deviation is presented as error bars

**Fig. 2:**
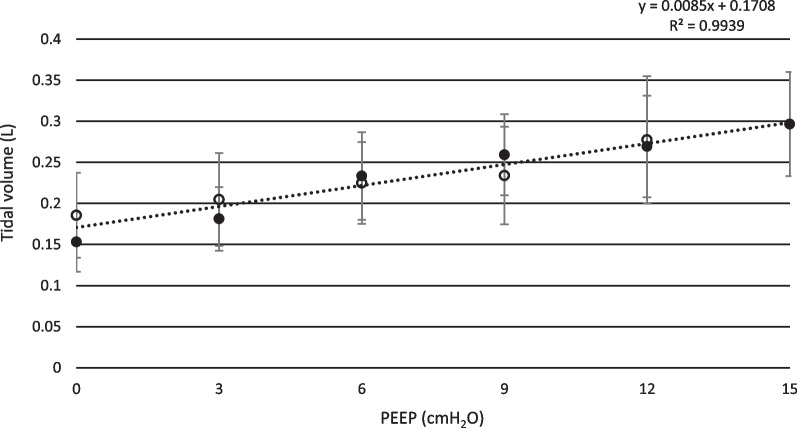
Tidal volume. Mean tidal volume is presented for incremental and decremental PEEP levels. Data was collected from a pneumotachograph connected to the endotracheal tube. Black dots represent incremental PEEP levels and transparent circles represent decremental PEEP levels. Standard deviation is presented as error bars

**Fig. 3:**
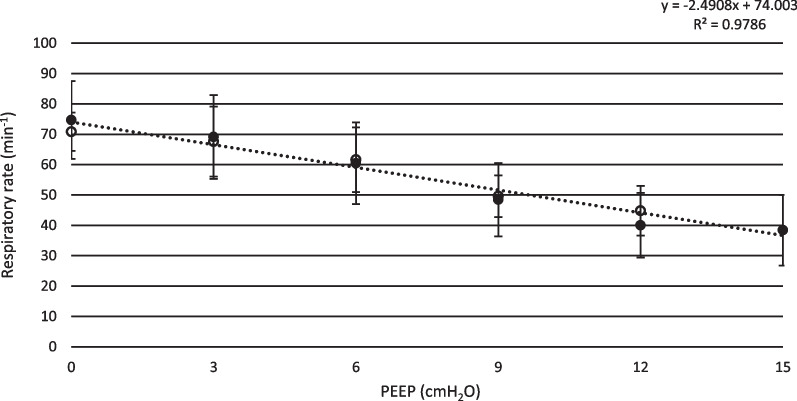
Respiratory rate. Mean respiratory rate is presented for incremental and decremental PEEP levels. During ZEEP, the animals were breathing very rapidly during both initiation and termination of the experiment. As PEEP was gradually increased, the respiratory rate decreased. Black dots represent incremental PEEP levels and transparent circles represent decremental PEEP levels. Standard deviation is presented as error bars. Trend line and correlation equation is presented

As PEEP was increased stepwise from 0 cmH_2_O to 15 cmH_2_O, mean peak pressure increased from 6.6 ± 2.8 cmH_2_O to 23.5 ± 1.7 cmH_2_O (t-test; p < 0.01). Transpulmonary pressure increased from 6.6 ± 3.2 cmH_2_O to 23.7 ± 2.1 cmH_2_O (t-test; p < 0.01), see Fig. 1. Tidal volume increased from 153 ± 36 ml to 297 ± 63 ml (t-test; p < 0.01), see Fig. 2, and respiratory rate decreased from 75 ± 13 min^−1^ to 38 ± 12 min^−1^ (p < 0.01 t-test), see Fig. 3. Calculated compliance increased from 14.5 ± 2.2 ml·cmH_2_O^−1^ to 26.9 ± 2.7 ml·cmH_2_O^−1^ (p < 0.01 t-test). Mean minimum PaO_2_ was 24.9 ± 12.3 mmHg, observed at PEEP 0.9 ± 1.4 cmH_2_O and mean maximum PaO_2_ was 76.2 ± 5.6 mmHg, observed at PEEP 13.8 ± 1.5 cmH_2_O (p < 0.01 t-test).

### Electric impedance tomography and tidal volume distribution

**Fig. 4:**
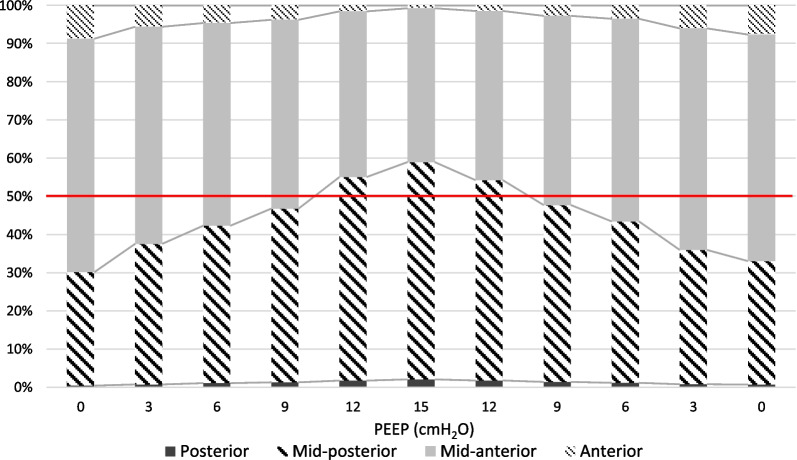
Tidal volume distribution. Tidal volume distribution acquired by EIT is presented for each ROI (anterior, mid-anterior, mid-posterior and posterior) in proportion to total change in all ROIs combined. The data represents the proportion of tidal volume being distributed to each of the lung regions, where the total tidal volume represents 100%. The red line visualizes regions where 50% of the total tidal volume is distributed. If the sum of posterior and mid-posterior ROI distribution exceeds 50%, the distribution is considered predominantly dependent

Stepwise increasing PEEP from 0 cmH_2_O to 15 cmH_2_O resulted in a mean anterior ∆Z decrease from 1.8 ± 1.2 to 0.31 ± 0.68 (p < 0.0033), a non-statistically significant mid-anterior ∆Z decrease from 13.0 ± 8.8 to 10.4 ± 4.0 (p > 0.0033), a mid-posterior increase in ∆Z from 6.3 ± 4.6 to 14.7 ± 5.2 (p < 0.0033) and a posterior ∆Z increase from 0.07 ± 0.09 to 0.54 ± 0.28 (p < 0.0033) when comparing PEEP 0 cmH_2_O to PEEP 15 cmH_2_O. The mentioned regional ∆Z values were calculated in proportion to the total ∆Z of the entire lung, allowing the estimation of the proportional distribution of tidal volume in the lung. As a result of increasing PEEP stepwise from 0 cmH_2_O to 15 cmH_2_O the proportional mean ∆Z in the anterior ROI decreased from 9 ± 3% to 1 ± 3% (p < 0.0033), in the mid-anterior ROI decreased from 61 ± 7% to 40 ± 8% (p < 0.0033), in the mid-posterior ROI increased from 30 ± 8 to 57 ± 8% (p < 0.0033) and in the posterior ROI increased from 0 ± 1% to 2 ± 1% (p < 0.0033), representing change in local tidal volume distribution, shown in Fig. 4.

**Fig. 5:**
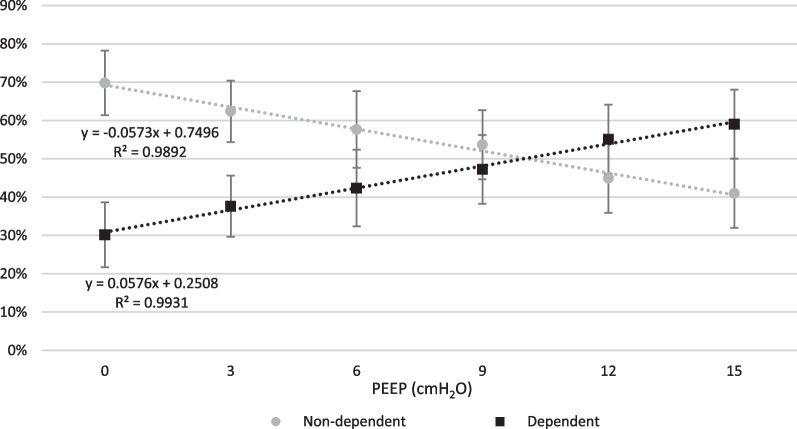
Dependent and non-dependent tidal volume distribution. Tidal volume distribution in the dependent and non-dependent regions are presented in relation to incremental PEEP levels. The dependent region consists of posterior and mid-posterior regions combined and non-dependent region consists of mid-anterior and anterior regions combined. During low PEEP levels the tidal volume is mainly distributed to the non-dependent region and as PEEP is gradually increased the tidal volume distribution is progressively shifted to dependent regions. During PEEP 9 cmH_2_O the distribution between dependent and non-dependent regions are closest to each other, representing the most homogeneous antero-posterior volume distribution. Further increasing PEEP caused an augmentation of dependent volume distribution dominance. Standard deviation is presented as error bars

**Fig. 6:**
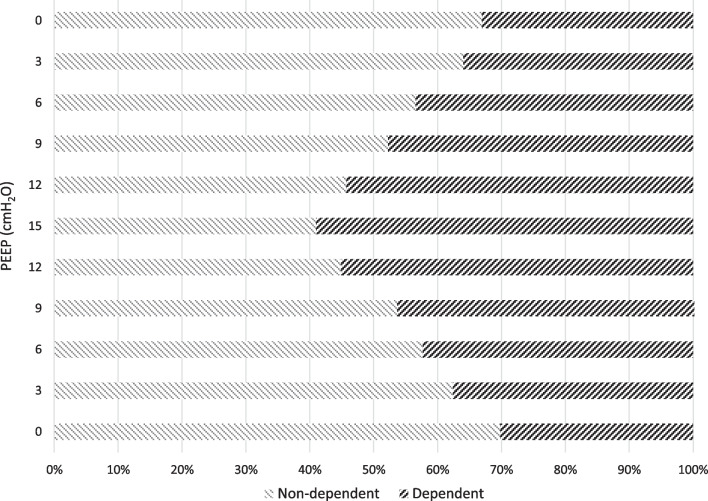
Dependent and non-dependent tidal volume distribution. The distribution of tidal volume distribution to non-dependent and dependent lung region is visualized in relation to total tidal volume distribution. The dependent region consists of posterior and mid-posterior regions combined and non-dependent region consists of mid-anterior and anterior regions combined

**Fig. 7:**
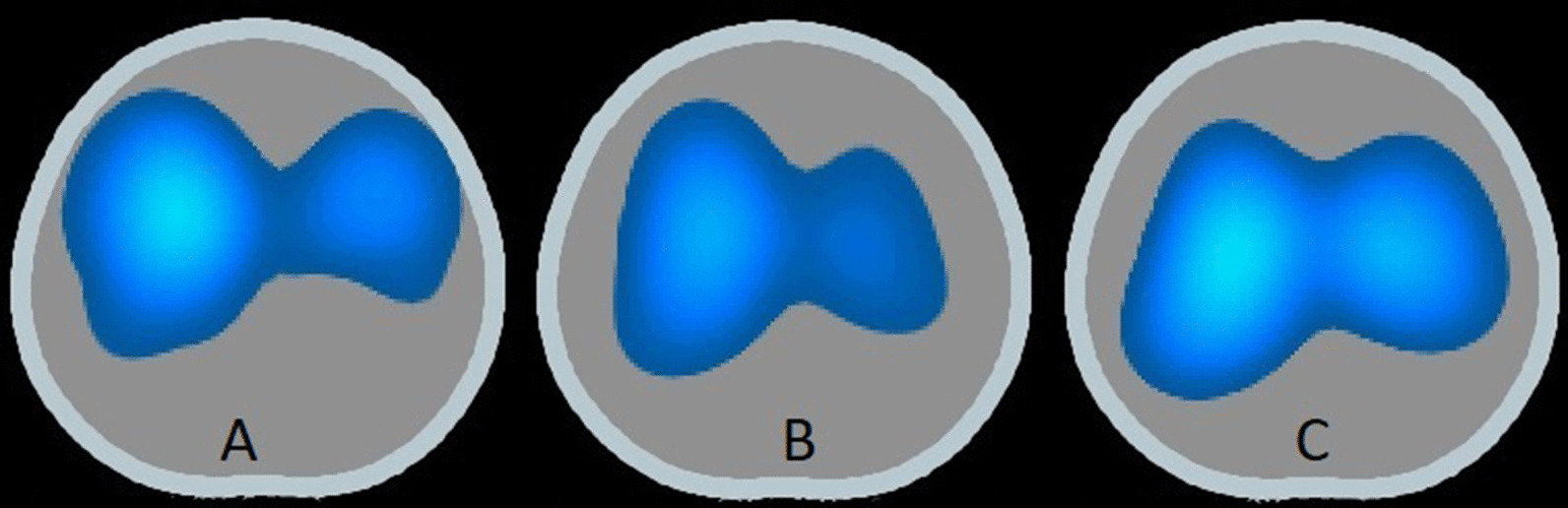
Representative EIT images. EIT images from one representative pig are shown for three different PEEP levels, 0 (**A**), 9 (**B**) and 15 cmH_2_O (**C**). The tidal volume is mainly distributed to the anterior lung regions during PEEP 0 cmH_2_O. As PEEP is gradually increased to 9 cmH_2_O and 15 cmH_2_O the tidal volume is shifted dorsally. During PEEP 15 cmH_2_O more than 50% of the tidal volume is distributed to the dorsal half of the lung

Ventilation was predominant in the non-dependent region of the lung (anterior and mid-anterior ROI) at PEEP 0 cmH_2_O and gradually shifted to the dependent region of the lung (mid-posterior and posterior ROI) when PEEP was increased stepwise. The ∆Z in the non-dependent lung in relation to total ∆Z decreased from 70 ± 8% to 41 ± 9% (p < 0.0033), and the ∆Z in the dependent lung in relation to total ∆Z increased from 30 ± 8% to 59 ± 9% (p < 0.0033), when PEEP stepwise was increased from 0 cmH_2_O to 15 cmH_2_O. The stepwise change in relative distribution of ∆Z, and thereby estimated tidal volume distribution, in dependent and non-dependent region is presented in Fig. 5, with linear regression analysis, and in Fig. 6. EIT images from one representative pig are presented in Fig. 7.

**Fig. 8:**
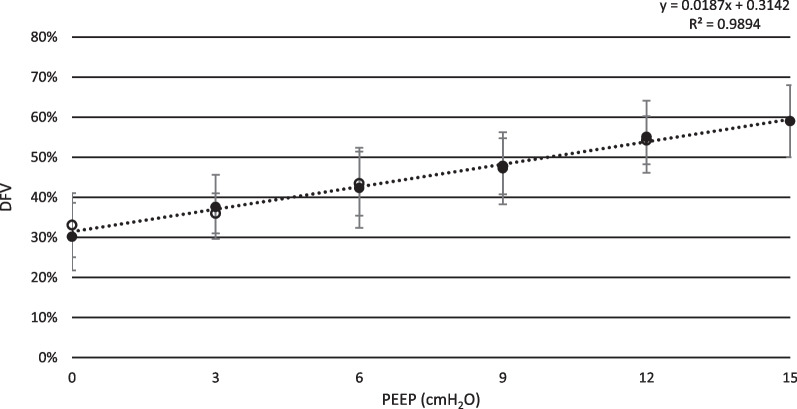
Dependent fraction of ventilation. The dependent fraction of ventilation (DFV) is shown for incremental and decremental PEEP levels. As PEEP is gradually increased there is a close to linear increase in DFV. When DFV is close to 50%, the distribution is considered homogenous. DVF > 50% indicates a predominantly dependent distribution. Black dots represent incremental PEEP levels and transparent circles represent decremental PEEP levels. There are no substantial differences between DFV originating from incremental or decremental PEEP levels. Standard deviation is presented as error bars

The dependent fraction of ventilation (DFV) increased from 30 ± 8% to 59 ± 9% (p < 0.0033) when comparing PEEP 0 cmH_2_O to PEEP 15 cmH_2_O. PEEP 9 cmH_2_O, having a DFV of 47%, is the PEEP level with a DFV nearest of 50% (PEEP 12 cmH_2_O had a DFV of 55%). There was a statistically significant increase in DFV from PEEP 0 cmH_2_O to PEEP 9 cmH_2_O (p < 0.0033) and significant difference between PEEP 9 cmH_2_O and PEEP 12 cmH_2_O (p < 0.0033). The DFV of each PEEP step is presented in Fig. 8.

## Discussion

To our knowledge this is the first study that analyses the relation between applied PEEP and ventilation distribution during NAVA. Our main finding is that the stepwise increase of PEEP generates a homogenizing effect on the distribution of tidal volume inside the lung, by progressively shifting tidal volume from non-dependent region to dependent lung regions. In our model this effect reached its maximum at PEEP 9 cmH_2_O while at the extrema of PEEP spectrum the difference in ventilation between the dependent and nondependent lung was augmented.

### Lung mechanics

Increasing PEEP raises peak airway pressure and the transpulmonary pressure, as end-expiratory airway pressure is increased. Simultaneously, the respiratory rate declines and tidal volume becomes higher. In other words, while PEEP gradually increases, the structure of breathing tends to change from a rapid and shallow to a slow and deep pattern, as previously shown in a similar set up [31]. Previous studies on the effect of PEEP on Vt have shown different results. In a human study, Passath et al. [32] demonstrated tidal volume preservation implementing a decremental PEEP titration during NAVA ventilation, differently from the results of the present study. However, some factors may explain this difference, such as the characteristics of their studied patients in terms of lung failure at the inclusion time or the grade of chest wall involvement (40% had either thoracic trauma or surgery). In our model the chest wall was intact and all the animals had a mild/moderate lung injury. Per se, the characteristics of the animal specific breathing reflexes may also have contributed to this difference. In this respect, our results are in line with the studies by Allo et al. [33], where phasic Edi and ∆Peso raised in response to PEEP increase in rabbits.

Furthermore, the PaO_2_ raised when PEEP was increased. The demonstrated effect of PEEP on oxygenation has been known for long [34] and comply well with similar animal experiments [35], and is presumably caused by the reduction of atelectasis-induced shunting and by alveolar recruitment [11].

### Electric impedance tomography

The difference in impedance (ΔZ) between end-expiration and end-inspiration represents the net effect of the ventilated volume (ΔV) on thorax impedance. The linear correlation between the ΔZ and ΔV [18, 19] can be used to estimate volume variation knowing impedance change.

In addition, EIT provides information about ventilation distribution [20–22]; in this study we have used this attribute by dividing the images in four equally spaced rectangular ROIs and observing which areas were subjected to variation of impedance due to redistribution of ventilation.

During ventilation at ZEEP, the highest tidal ΔZ is observed in the mid-anterior part of the lung. As PEEP is increased, the tidal ΔZ tends to move dorsally, to posterior ROIs. This indicates that a larger gas volume is reaching these areas. By analyzing the relative distribution of tidal volume while increasing PEEP from 0 to 15 cmH_2_O, it is possible to observe a significant decrease in tidal volume in anterior and mid-anterior lung sections associated to a parallel increase in tidal volume distribution to mid-posterior and posterior lung sections. The effect on shifting tidal volume dorsally may be further amplified by the increased tidal volume, as this tends to increase the distribution further posteriorly [36].

This indicates that increasing PEEP leads to a better ventilation, or even a recruitment, of dependent lung regions, rendering them active in the process of ventilation. This may potentially derive from the reduction of basal atelectasis or by the reduction of airway closure [37, 38].

Meanwhile, the increased PEEP causes a reduction of anterior non-dependent tidal volume, probably due to the overdistention of alveoli and the less favorable position on the compliance curve. These results conform well with prior studies [31] using the same experimental model, although performed using computed tomography.

**Fig. 9:**
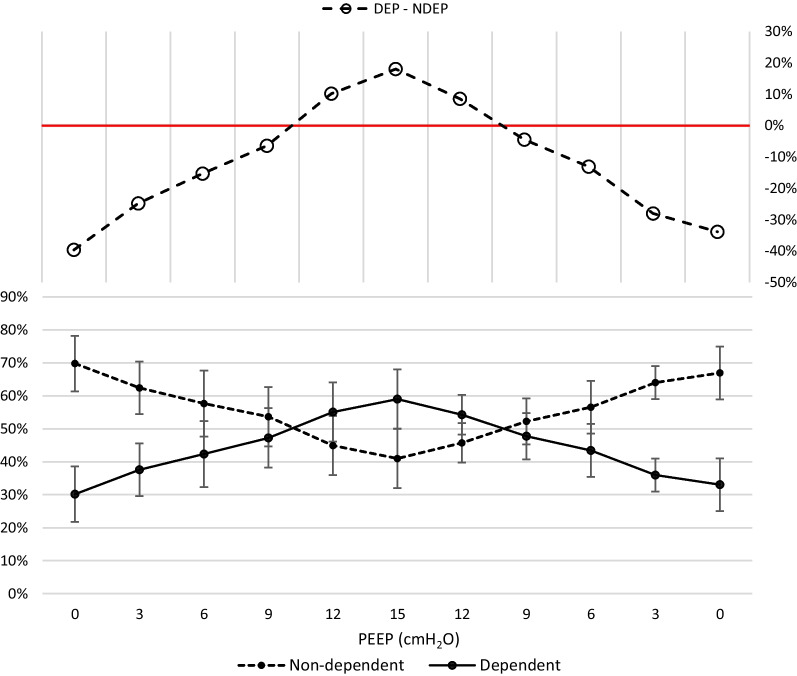
Difference between dependent and non-dependent tidal volume distribution. In the lower panel, the tidal volume distribution in the dependent and non-dependent region in percentage of total tidal volume is presented in relation to PEEP level. During the increase of PEEP the dependent portion of tidal volume distribution is increased. After PEEP 9 cmH_2_O is reached the line crosses, indicating that the dependent tidal volume distribution surpasses the non-dependent distribution. During the decremental PEEP phase the lines crosses prior to PEEP 9 cmH_2_O, indicating that after this PEEP level the distribution is returned to predominantly non-dependent. Standard deviation is presented as error bars. The balance between non-dependent and dependent distribution is further demonstrated in the upper panel where the difference of percentage points between non-dependent and dependent distribution is shown. When values are less than 0% the distribution is predominantly non-dependent. When values are close to 0%, indicated by the red line, the distribution is considered homogenous. When values exceed 0% the distribution is predominantly dependent

Importantly, increasing PEEP during NAVA ventilation can have a homogenizing effect on the distribution of tidal volume throughout the lung. Observing the curves of Fig. 9, it is possible to notice that the progressive shift of ΔZ has opposite direction in dependent and nondependent sections of the lung. In other terms, there is a point in which these two lines intersect, which represent the point in which there is the maximal homogeneity. In our animal model of ARDS ventilated with NAVA, the point of best homogeneity is at PEEP 9 cmH_2_O. After this point, the lines diverge again, meaning that the main areas of ventilation become the dependent ones.

As shown in this study, the use of tidal volume distribution offers a possible technique of PEEP titration during assisted SB, as compared to the titration method previously described by Costa et al. [20], useful during no flow conditions.

The tidal volume distribution differs amongst various ventilatory modes, and in the case of assisted spontaneous breathing, PSV and NAVA show distribution differences, related not only to the modality per se but also to the level of their application [39]. Correspondingly to these effects, there may be different effect of PEEP on tidal volume distribution amongst various ventilatory modes. PEEP has previously been shown to increase dependent tidal volume distribution during PSV in patients with ARDS [40]. The specific differences of tidal volume distribution amongst assisted as wells as controlled ventilatory modes as effect of PEEP needs further investigation. However, the effect of spontaneous breathing on posterior diaphragm and dependent lung has been known for long [41], and spontaneous efforts have previously been shown to increase the tidal volume distribution to dependent lung regions using EIT [42].

Similar results are possible to achieve by computing the so-called dorsal fraction of ventilation (DFV) as proposed by Brochard et al. [23]. By analyzing the proportion of the tidal volume distributing to the dependent lung, the homogeneity of tidal volume distribution may be estimated. The concept of DFV is similar to that of impedance ratio (IR) [43] and anterior–posterior ratio [44], able to detect lung recruitment and tidal volume distribution homogenization during PEEP increase. However, DFV is not to be confused with the concept of center of ventilation (COV), describing the center of distribution of tidal volume in relation to the chest geometry [22]. In this study, we show that, by increasing PEEP in NAVA ventilation, there is a gradual change from predominantly low dependent activity (DFV < 50%) to predominantly low non-dependent activity (DFP > 50%) and that the DFV closest to 50% is reached at PEEP 9 cmH_2_O. These findings are in line with the results from Katira et al. [25], in which they use a similar experimental set up, but studying controlled ventilation. The results show similar effect of non-dependent to dependent ventilation shift with a DFV ≈ 50% at PEEP 9 cmH_2_O, indicating that the effect of PEEP on tidal volume distribution may be similar when using controlled ventilation and NAVA, although further studies are needed.

Ultimately, the PEEP level must be set aiming at either reducing dependent atelectasis or reducing non-dependent overinflation, in relation to the present DFV and the clinical situation. The goal of balancing these impairing phenomena may be achieved by aiming at a DFV of 50%, in which the level of stress is more uniformly distributed throughout the lung.

To titrate the PEEP in order to get the most homogeneous distribution of lung volumes, the EIT is exceptionally helpful.

### Clinical implication

The findings in this paper suggest that during NAVA ventilation, and potentially other assisted breathing modes, EIT can be used for continuous monitoring of tidal volume distribution. Thereby, the technique can potentially be used for PEEP level selection and titration, guided by either signs of dependent lung recruitment and non-dependent hyperinflation, or by the use of dependent fraction of ventilation. The results in this paper stress the effect and the importance of carefully choosing the PEEP level in spontaneously breathing subjects, as the consequences on lung ventilation are considerable.

### Limitations

The present study has different limitations. An animal model of mild ARDS was used, by inducing lung injury through lavage and pulmonary suctioning. This may vary from the authentic condition seen in human patients, and may possibly be more recruitable. In addition, more severe ARDS may be less suitable for the use of assisted spontaneous breathing. Regarding the use of animals, it is important to notice that respiratory drive and respiratory reflexes may differ amongst species. This indicates that the results may not be directly transposed to the clinical settings nor be extrapolated to severe ARDS. Furthermore, the PEEP levels were not randomized. However, this was intentional as incremental and decremental PEEP levels could be compared, testing for differences amongst incremental and decremental levels indicating time dependent reduction of effect of lung injury or a permanent recruitment effect of high PEEP, however this effect was not observed. In the present study, the analysis of tissue histology and inflammatory markers was not performed although it could have contributed to collect additional information about the studied model.

As previously shown, increased Vt shifts tidal volume distribution dorsally. Hence, in this study showing increased Vt as an effect of increasing PEEP, a contributing effect of Vt changes on the tidal volume distribution cannot be ruled out. However, DFV shows statistically significant differences between PEEP levels only having minor differences in tidal volume, indicating that Vt alone is not responsible of tidal volume distribution changes.

Furthermore, this study evaluates the effect of PEEP on lung mechanics and tidal volume distribution and may not conform to other modes of assisted spontaneous breathing.

Regarding EIT analysis, there is an important phenomenon resulting from the effect of increasing PEEP that needs to be noted. As PEEP is increased from 0 to 15 cmH_2_O, the lung is expanded inferiorly, shifting the parenchyma adjacent to the EIT belt. However, in relation to the dimension of the lungs of the pigs used, EIT generates images pertaining to approximately half of the cranio-caudal extension of the lung [45]. From previous studies on porcine models of the same size and comparable lung inflation (see Perchiazzi et al., supplementary material [4]) it is possible to conclude that EIT maintains *in view* the same lung structures throughout the increasing PEEP sequence used in the present paper. For this reason, any possible flaws deriving for the downward shift of lung parenchyma were considered not affecting the results of our analysis.

## Data Availability

The datasets analysed during the current study are available from the corresponding author on reasonable request.
